# Variation at the *TRIM11* locus modifies progressive supranuclear palsy phenotype

**DOI:** 10.1002/ana.25308

**Published:** 2018-09-15

**Authors:** Edwin Jabbari, John Woodside, Manuela M. X. Tan, Maryam Shoai, Alan Pittman, Raffaele Ferrari, Kin Y. Mok, David Zhang, Regina H. Reynolds, Rohan de Silva, Max‐Joseph Grimm, Gesine Respondek, Ulrich Müller, Safa Al‐Sarraj, Stephen M. Gentleman, Andrew J. Lees, Thomas T. Warner, John Hardy, Tamas Revesz, Günter U. Höglinger, Janice L. Holton, Mina Ryten, Huw R. Morris

**Affiliations:** ^1^ Department of Clinical and Movement Neurosciences Institute of Neurology, University College London London United Kingdom; ^2^ Department of Neurodegenerative Disease Institute of Neurology, University College London London United Kingdom; ^3^ Reta Lila Weston Institute UCL Institute of Neurology London United Kingdom; ^4^ Department of Translational Neurodegeneration German Center for Neurodegenerative Diseases (DZNE); Department of Neurology, Technical University of Munich; Munich Cluster for Systems Neurology SyNergy Munich Germany; ^5^ Institute for Human Genetics Justus Liebig University Giessen Germany; ^6^ MRC London Neurodegenerative Diseases Brain Bank, Institute of Psychiatry, King's College London London United Kingdom; ^7^ Multiple Sclerosis and Parkinson's UK Brain Bank, Division of Brain Sciences, Imperial College London, Hammersmith Hospital Campus London United Kingdom; ^8^ Queen Square Brain Bank for Neurological Disorders, UCL Institute of Neurology London United Kingdom

## Abstract

**Objective:**

The basis for clinical variation related to underlying progressive supranuclear palsy (PSP) pathology is unknown. We performed a genome‐wide association study (GWAS) to identify genetic determinants of PSP phenotype.

**Methods:**

Two independent pathological and clinically diagnosed PSP cohorts were genotyped and phenotyped to create Richardson syndrome (RS) and non‐RS groups. We carried out separate logistic regression GWASs to compare RS and non‐RS groups and then combined datasets to carry out a whole cohort analysis (RS = 367, non‐RS = 130). We validated our findings in a third cohort by referring to data from 100 deeply phenotyped cases from a recent GWAS. We assessed the expression/coexpression patterns of our identified genes and used our data to carry out gene‐based association testing.

**Results:**

Our lead single nucleotide polymorphism (SNP), rs564309, showed an association signal in both cohorts, reaching genome‐wide significance in our whole cohort analysis (odds ratio = 5.5, 95% confidence interval = 3.2–10.0, *p* = 1.7 × 10^−9^). rs564309 is an intronic variant of the tripartite motif‐containing protein 11 (*TRIM11*) gene, a component of the ubiquitin proteasome system (UPS). In our third cohort, minor allele frequencies of surrogate SNPs in high linkage disequilibrium with rs564309 replicated our findings. Gene‐based association testing confirmed an association signal at *TRIM11*. We found that TRIM11 is predominantly expressed neuronally, in the cerebellum and basal ganglia.

**Interpretation:**

Our study suggests that the *TRIM11* locus is a genetic modifier of PSP phenotype and potentially adds further evidence for the UPS having a key role in tau pathology, therefore representing a target for disease‐modifying therapies. Ann Neurol 2018;84:485–496

Progressive supranuclear palsy (PSP) is a progressive neurodegenerative condition and the most common cause of atypical parkinsonism, with an estimated prevalence of 5 to 7 per 100,000.^1^ The pathology of PSP is centered on the structural microtubule‐associated protein tau, encoded by the *MAPT* gene located on chromosome 17. In PSP, there is neuronal and glial accumulation of hyperphosphorylated fibrillary aggregates of 4‐repeat predominant tau. The pathological hallmarks of PSP include a high density of neurofibrillary tangles and neuropil threads in the basal ganglia and brainstem along with tau‐positive tufted astrocytes.^2^

Richardson syndrome (RS) is the most common clinical phenotype related to PSP pathology. It was first described by Steele, Richardson, and Olszewski as an L‐dopa–unresponsive akinetic–rigid syndrome with falls, a vertical supranuclear gaze palsy, and dementia.^3^ Previous studies looking at the natural history of RS have shown that the mean age of disease onset is 65 to 67 years, and the median disease duration is 6 to 7 years.^4^ In addition, a clinical diagnosis of RS has been shown to be highly predictive of underlying PSP pathology,^5^ and the diagnosis of this form of PSP was operationalized in the NINDS–Society for Progressive Supranuclear Palsy criteria.^6^

We and others have identified alternative clinical phenotypes^7^, ^8^ related to PSP pathology in relatively small case series. This led to the description of 2 distinct PSP non‐RS clinical phenotypes by Williams and colleagues, PSP‐parkinsonism (PSP‐P)^9^ and pure akinesia with gait freezing (PAGF).^10^ PSP‐P and PAGF have a similar age of disease onset to RS, clinically resemble RS in the latter stages of disease, and have a significantly longer mean disease duration (PSP‐P = 9 years, PAGF = 13 years). The basis for this clinical variation related to a core pathology is unknown. PSP clinical subtypes have been related to the regional distribution and severity of pathogenic tau accumulation and neuronal loss.^11^ Although postmortem remains the gold standard for diagnosing PSP, recent publication of new diagnostic criteria from the Movement Disorder Society (MDS) PSP study group^12^ highlight the presence of PSP‐P and PAGF along with other PSP clinical phenotypes relating to underlying PSP pathology including PSP‐corticobasal (PSP‐CBS)^13^ and PSP‐frontal (PSP‐F) subtypes.^14^

A recent comprehensive genome‐wide association study (GWAS) involving 1,114 pathologically confirmed PSP cases and 3,247 controls was carried out to identify common risk variants for PSP. Single nucleotide polymorphisms (SNPs) that passed a significance cutoff point of *p* ≤ 10^−3^ were subsequently genotyped in a validation cohort that consisted of 1,051 clinically diagnosed PSP cases and 3,560 controls. Loci at MAPT (H1 haplotype and H1c sub‐haplotype), MOBP, STX6, and EIF2AK3 were associated with PSP.^15^

Differences in the clinicopathological phenotypes of tauopathies (including Alzheimer disease) may relate to differences in the strain properties of toxic tau species.^16^ However, here we use a large clinicopathological cohort based on the latest MDS diagnostic criteria to show that the clinical phenotype of PSP relates in part to genetic variants that may determine regional susceptibility.

## Subjects and Methods

### *Study Design and Participants*

All patients gave written informed consent for the use of their medical records and blood/brain tissue for research purposes, including the analysis of DNA. Patients with a neuropathological diagnosis of PSP were identified from the following UK brain banks: MRC London Neurodegenerative Diseases Brain Bank (Research Ethics Committee reference 08/MRE09/38 + 5), Multiple Sclerosis and Parkinson's UK Brain Bank, London (Research Ethics Committee reference 08/MRE09/31 + 5), and Queen Square Brain Bank (the brain donor program was approved by a London Multi‐Centre Research Ethics Committee, and tissue is stored for research under a license from the Human Tissue Authority, No. 12198). The year of death for cases ranged from 1998 to 2017.

Patients with a clinical diagnosis of a PSP syndrome were identified from the Progressive Supranuclear Palsy Cortico‐Basal Syndrome Multiple System Atrophy Longitudinal UK (PROSPECT‐UK) study, a longitudinal study of patients with atypical parkinsonian syndromes undergoing serial clinical, imaging, and biomarker measures (Queen Square Research Ethics Committee 14/LO/1575). Cases were recruited between 2015 and 2017. A subset of these patients also underwent postmortem neuropathological diagnosis at the Queen Square Brain Bank.

### *Phenotyping of Cases*

Retrospective clinical notes review of all neuropathological PSP cases was performed to extract the following demographic and clinical information: gender, age at motor symptom onset, date of motor symptom onset, and date of death. This information was used to calculate the total disease duration (defined as date of death − date of motor symptom onset). Cases that did not have the above clinical information available were excluded from the study. Exclusion criteria used in the MDS diagnostic criteria were not considered, as the presence of alternative diseases would have been identified at postmortem. Using the MDS diagnostic criteria, each case was assigned an initial and final clinical phenotype.^12^ This was based on the clinical features documented in clinical letters in the first 3 years from motor symptom onset and the clinical features documented in clinical letters in the last 2 years of life. We focused on 3 clinical phenotypes of interest: RS, PSP‐P, and PAGF; and only assigned these phenotypes if their corresponding “probable” criteria were fulfilled. Cases were assigned a diagnosis of “unclassified” if there was insufficient evidence from the clinical notes to assign one of the phenotypes of interest. In cases where there was an overlap of clinical phenotype features, a consensus decision was made to assign the most appropriate clinical phenotype. The same clinical data as above were collected on clinically diagnosed PSP cases using their PROSPECT‐UK study clinical assessments. To ensure accuracy in assigning a phenotype, living subjects were only included if their latest clinical assessment was carried out at least 3 years after motor symptom onset. In addition, cases were excluded from analyses if they had the presence of any MDS diagnostic exclusion criteria or if they fulfilled both MDS criteria for one of our PSP phenotypes of interest as well as Armstrong criteria for probable PSP‐CBS, as these subjects may have underlying corticobasal degeneration pathology.^17^

### *Genotyping and Quality Control*

All pathologically diagnosed cases had DNA extracted from frozen brain tissue (cerebellum or frontal cortex). Subsequently, DNA samples from all cases underwent genotyping using the Illumina (San Diego, CA) NeuroChip.^18^ Standard genotype data quality control steps were carried out as per Reed et al,^19^ including a principal component analysis (PCA) to exclude all non‐European subjects. All cases were screened for known *MAPT, LRRK2*, and *DCTN1* mutations covered by the NeuroChip. SNP imputation was carried out on our NeuroChip data using the Sanger Imputation Service to produce a final list of common (minor allele frequency ≥ 1%) variants for analyses. Imputed SNP positions were based on Genome Reference Consortium Human 37/human genome version 19 (GRCh37/hg19). Standard quality control steps taken for SNP imputation were carried out as per Reed et al.^19^

To confirm the validity of our NeuroChip genotyping and imputation, a subset of both directly genotyped and imputed SNPs underwent regenotyping using the LGC KASP genotyping service for coverage of significant regions in association.

### *Statistical Analyses*

All statistical analyses were carried out using Plink v1.9 and images generated using R v3.3.2 and LocusZoom.

By dividing the whole cohort into RS and non‐RS (combined PSP‐P and PAGF) groups based on their initial clinical phenotype, group comparisons of clinical features were carried out using *t* tests. In addition, the RS and non‐RS group minor allele frequencies (MAFs) of all PSP case–control GWAS risk variants were extracted from our imputed data.

### *Logistic Regression GWAS*

A logistic regression GWAS was performed on our imputed data to compare RS and non‐RS groups. Based on their assigned initial clinical phenotypes, non‐RS subjects were defined as “cases” and RS subjects were defined as “controls.” The regression model used gender, age at motor symptom onset, study site of subject recruitment, and the first 2 principal components as covariates. This analysis was first carried out on our pathological cohort and then subsequently on our clinical cohort before combining datasets to carry out a whole cohort analysis. The Bonferroni correction for multiple SNP testing was used to set the genome‐wide significance *p* value threshold at 9 × 10^−9^. The whole cohort GWAS analysis was used to generate Manhattan and regional association plots.

All significant SNPs from our association analysis were assessed for their MAFs in European controls. This data was acquired from the Genome Aggregation Database (http://gnomad.broadinstitute.org), which is based on data from ∼120,000 exome sequences and ∼15,500 whole genome sequences from unrelated individuals.

All significant SNPs from our association analysis were assessed for their level of significance in phase 1 of the original PSP case–control GWAS^15^ using publicly available data at the National Institute on Aging Genetics of Alzheimer's Disease Data Storage Site (http://www.niagads.org).

### *PSP Case–Control GWAS Validation Cohort*

A separate subset of 100 pathologically confirmed PSP cases from phase 1 of the original PSP case–control GWAS had in‐depth phenotype data available to assign an initial clinical phenotype according to the MDS criteria, as per our study methods. These cases had undergone genotyping using the Illumina Human 660W‐Quad Infinium Beadchip with standard data quality control steps taken, including a PCA to exclude non‐Europeans. RS and non‐RS group MAFs for directly genotyped SNPs that were significant in our phenotype GWAS were extracted to further validate our findings.

### *Gene‐Based Association Testing*

Gene‐level *p* values were calculated using MAGMA v1.06 as outlined in de Leeuw et al.^20^ MAGMA tests the joint association of all SNPs in a gene with the phenotype while accounting for linkage disequilibrium (LD) between SNPs. This presents a powerful alternative to SNP‐based analyses, as it reduces the multiple testing burden and thus increases the possibility of detecting effects consisting of multiple weaker associations.^20^ SNPs were mapped to genes using National Center for Biotechnology Information (NCBI) definitions (GRCh37/hg19, annotation release 105); only genes in which at least one SNP mapped were included in downstream analyses. These were run both with and without a window of 35kb upstream and 10kb downstream of each gene, as most transcriptional regulatory elements fall within this interval.^21^ Furthermore, the major histocompatibility complex region was excluded. The gene *p* value was computed based on the mean association statistic of SNPs within a gene, with genome‐wide significance set to *p* < 2.74 × 10^−6^, and LD was estimated from the European subset of 1000 Genomes Phase 3.

### *Whole Exome Sequencing*

Sixty‐nine cases from our pathological cohort had previously undergone whole exome sequencing (WES) using the Illumina Truseq Capture in Illumina HiSeq platform. These data were used to look for the presence of rare coding variants in genes of interest to arise from our GWAS. Read data were aligned to hg19 by use of novoalign (v3.02.04) and indexed bam files were deduplicated of polymerase chain reaction artifacts by use of Picard Tools MarkDuplicates. The Genome Analysis Toolkit was then used to perform all subsequent steps according to their good practice; local realignments around possible indels and variant calling were conducted with HaplotypeCaller. Variants were filtered by use of variant quality score recalibration (truth tranche = 99.0%). In addition, hard‐filtering based on low‐depth and low‐genotype quality was performed. Annotation was performed by use of Annovar software.

### *Assessment of Gene Expression*

Gene expression profiles were assessed using publicly available BRAINEAC (http://www.braineac.org),^22^ GTEx (http://www.gtexportal.org), and Allen Brain Atlas (http://www.brain-map.org)^23^ Web‐based resources.

The BRAINEAC database contains brain tissues from 134 healthy controls from the following brain regions: frontal cortex, temporal cortex, parietal cortex, occipital cortex, hippocampus, thalamus, putamen, substantia nigra, medulla, cerebellum, and white matter. RNA isolation and processing of brain samples were performed and analyzed using Affymetrix (Santa Clara, CA) Exon 1.0 ST arrays. The GTEx database consists of 8,555 samples from 53 tissues (including 13 brain regions) of 544 donors for which RNAseq was conducted. The GTEx Project was supported by the Common Fund of the Office of the Director of the National Institutes of Health, and by the National Cancer Institute, National Human Genome Research Institute, National Heart, Lung, and Blood Institute, National Institute on Drug Abuse, National Institute of Mental Health, and NINDS. The data used for the analyses described in this article were obtained from the GTEx Portal on April 31, 2018. The Allen Human Brain Atlas database contains microarray data from 8 neuropathologically normal individuals of varying ethnicity. Microarray data were generated using the Agilent Technologies (Santa Clara, CA) 4 × 44 Whole Human Genome array and covers ∼62,000 gene probes per profile and ∼150 brain regions.

Gene expression at the cellular level in the brain was analyzed using RNAseq data from the Brain RNA‐Seq database (http://www.brainrnaseq.org/) as per Zhang et al.^24^ Of note, these data were generated from healthy temporal lobe samples that were resected from 14 patients to gain access to deeper epileptic hippocampi. The number of different cell types obtained from these samples were as follows: mature astrocyte, n = 12; microglia, n = 3; neuron, n = 1; oligodendrocyte, n = 3. We also used single cell RNA‐Seq data provided by DropViz (http://www.dropviz.org), which provides gene expression on 690,000 individual cells derived from 9 different regions of the adult mouse brain.^25^

### *Colocalization Analyses*

To evaluate the probability that the same causal SNP was responsible for modifying the phenotype of PSP and modulating gene expression, we performed the Coloc method described by Giambartolomei et al,^26^ using our GWAS summary statistics coupled with expression quantitative trait loci (eQTLs) from Braineac and GTEx. GTEx eQTLs included those originating from all brain regions. We restricted analyses to genes within 1Mb of the significant region of interest (*p* < 5 × 10^−8^) and ran coloc.abf with default priors. We considered tests with a Posterior probability of hypothesis 4 (PPH4) ≥ 0.75 to have strong evidence for colocalization.

## Results

A total of 497 subjects were included for analyses. Their clinical features are summarized by cohort and disease group in Table 1. Forty‐four subjects were deemed unclassifiable and therefore not included in subsequent analyses.

**Table 1:** Clinical Features of Subjects Included in Genotype–Phenotype Analyses

Feature	Pathological Cohort		Clinical Cohort		Whole Cohort	
	RS	Non‐RS	RS	Non‐RS	RS	Non‐RS
Subjects, n	230	76, PSP‐P = 60, PAGF = 16	137	54, PSP‐P = 42, PAGF = 12	367	130, PSP‐P = 102, PAGF = 28
Male, %	60.0	53.9	57.7	51.9	59.1	53.1
Age at motor symptom onset, yr, mean, range, SD	68.9, 49–89, 7.4	65.9, 46–86, 8.8	66.5, 51–87, 6.8	65.3, 54–82, 6.9	68.1,^a^, ^b^ 49–89, 7.3	65.6,^a^, ^b^ 46–86, 8.0
Final/current clinical phenotype	RS = 230	RS = 71, PSP‐P = 4, PAGF = 1	RS = 137	RS = 28, PSP‐P = 20, PAGF = 6	RS = 367	RS = 99, PSP‐P = 24, PAGF = 7
Mean disease duration in deceased subjects, yr, mean, range, SD	5.9, 1.9–15.9, 1.9	10.7, 2.2–16.3, 2.9	5.6, 2.4–13.5, 2.2	9.2, 8.1–10.8, 1.2	5.8,^b^, ^c^ 1.9–15.9, 1.9	10.6,^b^, ^c^ 2.4–13.5, 2.8
Subjects undergoing postmortem, n (% with a pathological diagnosis of PSP)	230 (100)	76 (100)	10 (100)	1 (100)	240 (100)	77 (100)

RS/non‐RS status is based on initial clinical phenotype.

a No statistically significant difference between RS and non‐RS groups.

b No statistically significant difference between pathological and clinical cohorts.

c Statistically significant (*p* < 0.05) difference between RS and non‐RS groups using Welch *t* test.

PAGF = pure akinesia with gait freezing; PSP = progressive supranuclear palsy; PSP‐P = PSP‐parkinsonism; RS = Richardson syndrome; SD = standard deviation.

An initial screen of our genotype data revealed similar MAFs for risk variants identified in the PSP case–control GWAS (Table 2).

**Table 2:** Comparison of PSP Risk Variant Status between PSP Case–Control GWAS and PSP Phenotype GWAS Data

Chr. band	SNP Position, bp	Gene	PSP Case–Control GWAS		PSP Phenotype GWAS	
			MAF in Healthy Controls	MAF in PSP	MAF in RS	MAF in Non‐RS
1q25.3	rs1411478, 180,962,282	*STX6*	0.42	0.50	0.44^a^	0.43
2p11.2	rs7571971, 88,895,351	*EIF2AK3*	0.26	0.31	0.34^a^	0.30
3p22.1	rs1768208, 39,523,003	*MOBP*	0.29	0.36	0.32^a^	0.32
17q21.31	rs8070723, 44,081,064	*MAPT* (H1 haplotype)	0.23	0.05	0.05^a^	0.05
	rs242557, 44,019,712	*MAPT* (H1c subhaplotype)	0.35	0.53	0.44^a^	0.49

PSP case–control GWAS data taken from Hoglinger et al.^15^

a No statistically significant difference between RS and non‐RS groups using Fisher exact test.

Chr. = chromosome; GWAS = GWAS; MAF = minor allele frequency; PSP = progressive supranuclear palsy; RS = Richardson syndrome; SNP = single nucleotide polymorphism.

**Figure 1:**
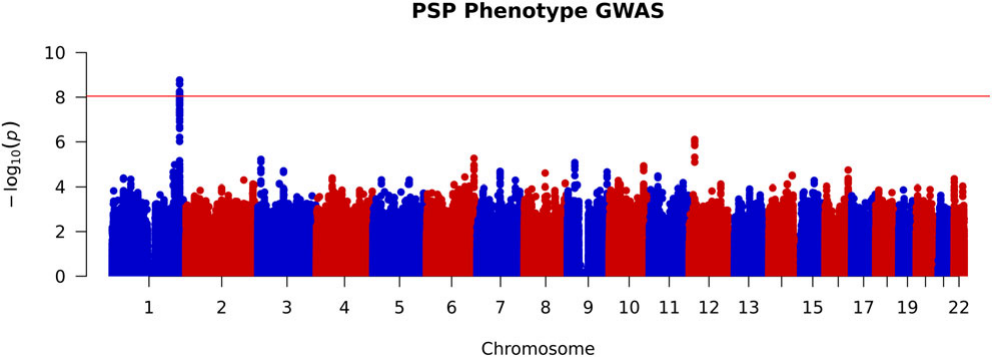
Manhattan plot of whole cohort Richardson syndrome (RS) versus non‐RS association analysis, highlighting genome‐wide significance at chromosome 1. The red line indicates the threshold for genome‐wide significance (*p* < 9 × 10^−9^). GWAS = genome‐wide association study; PSP = progressive supranuclear palsy.

In addition, none of our cases carried known pathogenic variants covered by the NeuroChip for the following genes: *MAPT* (40 variants), *LRRK2* G2019S (1 variant), and *DCTN1* (12 variants). After SNP imputation and data quality control, 6,215,948 common variants were included in our analysis. Assigning non‐RS subjects as “cases” and RS subjects as “controls,” we applied a logistic regression association analysis using gender, age at motor symptom onset, study site of subject recruitment, and the first 2 principal components as covariates. We first carried out this analysis using data from our pathological cohort and then validated our findings using data from our independent clinical cohort before combining both datasets for a whole cohort analysis. The whole cohort analysis revealed 27 SNPs, all located on chromosome 1, which passed the threshold for genome‐wide significance (*p* < 9 × 10^−9^). These results are summarized in Figure 1. Population stratification was not evident in our cohort, as non‐European subjects were excluded from analyses as part of our genotype data quality control. This was further confirmed by obtaining a genomic inflation factor (lambda) value of 1.05. A further locus on chromosome 12 approached genome‐wide significance with the lead SNP (rs621042) *p* = 7.8 × 10^−7^.

**Figure 2:**
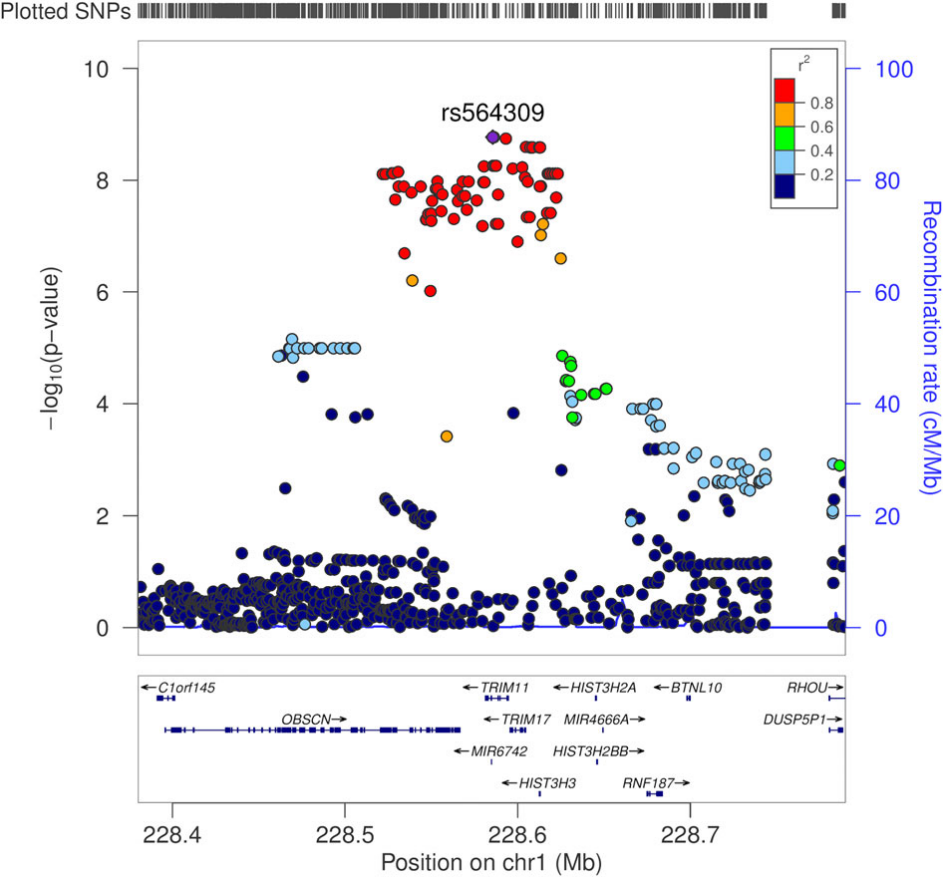
Regional association plot of Richardson syndrome (RS) versus non‐RS association analysis using imputed single nucleotide polymorphism (SNP) data, implicating the chromosome 1q42.13 locus and identifying rs564309, an intronic variant of *TRIM11*, as our lead SNP. SNP positions, recombination rates, and gene boundaries are based on GRCh37/hg19.

An in‐depth analysis of our significant SNPs reveal that they are all in high LD with each other (as defined by *r* ^2^ > 0.80) and are all located at the chromosome 1q42.13 locus. A regional association plot (Fig 2) reveals that our lead SNP, rs564309, is an intronic variant located between exons 3 and 4 of the tripartite motif‐containing protein 11 (*TRIM11*) gene. Alongside our directly genotyped lead SNP, the imputation quality score for imputed significant SNPs ranged from 0.96 to 1. Ninety‐six cases from our pathological cohort underwent regenotyping for 8 SNPs (rs564309, rs35670307, rs12065815, rs10158354, rs3795811, rs6426503, rs138782220, and rs7555298) that span the significant chromosome 1q42.13 locus. Three of the 8 SNPs, including our lead SNP, were originally directly genotyped via the NeuroChip, whereas the remaining 5 SNPs were originally imputed in our dataset. The *p* value of these SNPs in our whole cohort GWAS ranged from 1.7 × 10^−9^ to 7.3 × 10^−5^. The results of this regenotyping run showed 100% concordance with our original NeuroChip and imputation data.

Furthermore, 3 of the significant SNPs in LD with rs564309 are nonsynonymous (missense) coding variants of the Obscurin (*OBSCN*) gene. *OBSCN* is mainly expressed in skeletal muscle and may have a role in the organization of myofibrils during assembly as well as mediating interactions between the sarcoplasmic reticulum and myofibrils.^27^ Related diseases include fibromuscular dysplasia and hypertrophic obstructive cardiomyopathy.

The association statistics for rs564309, and the most significant flanking SNPs located at neighboring genes within the chromosome 1q42.12 locus, are summarized below (Table 3). The MAF of these SNPs was shown to be 0.10 in healthy European controls in the gnomAD database.

**Table 3:** RS versus Non‐RS Association Statistics for rs564309, and the Most Significant Flanking SNPs Located at Neighboring Genes, in Pathological, Clinical, and Whole Cohorts, Respectively

SNP Position, bp	Gene	Pathological Cohort			Clinical Cohort			Whole Cohort	
		MAF in RS	MAF in Non‐RS	OR (95% CI)	MAF in RS	MAF in Non‐RS	OR (95% CI)	OR (95% CI)	*p* a
rs564309, 228,585,562	*TRIM11*	0.04	0.19	6.25^b^ (3.12–12.5)	0.04	0.16	4.76^b^ (1.96–12.5)	5.55 (3.22–10.0)	1.7 × 10^−9^
rs61827276, 228,597,130	*TRIM17*	0.04	0.18	5.88^b^ (2.78–12.5)	0.04	0.16	5.55^b^ (2.17–14.3)	5.55 (3.12–10.0)	6.2 × 10^−9^
rs61825312, 228,530,748	*OBSCN*	0.04	0.19	5.88^b^ (2.86–12.5)	0.04	0.16	4.35^b^ (1.78–11.1)	5.26 (2.94–9.09)	7.1 × 10^−9^
rs2230656, 228,612,838	*HIST3H3*	0.06	0.23	4.35^b^ (2.32 –8.33)	0.06	0.20	3.70^b^ (1.75–8.33)	4.00 (2.50–6.67)	1.3 × 10^−8^

SNP positions are based on GRCh37/hg19.

a Probability value in whole cohort analysis.

b *p* > 9 × 10^−9^.

CI = confidence interval; MAF = minor allele frequency; Non‐RS = combined PSP‐parkinsonism and pure akinesia with gait freezing group; OR = odds ratio; PSP = progressive supranuclear palsy; RS = PSP‐Richardson syndrome group; SNP = single nucleotide polymorphism.

To explore the impact of inadvertently including Parkinson disease (PD) cases in our clinically diagnosed non‐RS group, we referred to genotyping data from 484 European clinically diagnosed PD cases that were genotyped alongside our PSP cases and had undergone the same quality control steps outlined above. We found that the MAF of rs564309 in PD cases was 7%, similar to the MAF in healthy controls and considerably lower than the MAF in our non‐RS group.

When referring to publicly available *p* value data from phase 1 of the original PSP case–control GWAS, we found that none of our significant SNPs reached even nominal significance (*p* < 0.05). One hundred European pathologically confirmed PSP cases from this GWAS underwent retrospective phenotyping according to the MDS diagnostic criteria using available clinical notes. Of those, 83 cases fulfilled probable criteria for initial clinical phenotypes of relevance to this study (PSP‐RS, n = 45; PSP‐P, n = 38). rs1188473, a SNP that was directly genotyped in the case–control GWAS, in high LD with our lead SNP (*r* ^2^ 1.0) and found to be significant in our phenotype GWAS (*p* = 2.6 × 10^−9^), was shown to have similar MAFs when comparing the GWAS datasets in both RS (4% vs 6%) and non‐RS (16% vs 16%) groups, therefore further validating our findings.

Analysis of WES data from 65 subjects (49 RS, 16 non‐RS) within our pathological cohort did not identify any nonsynonymous coding variants in *TRIM11* or *TRIM17* genes.

**Figure 3:**
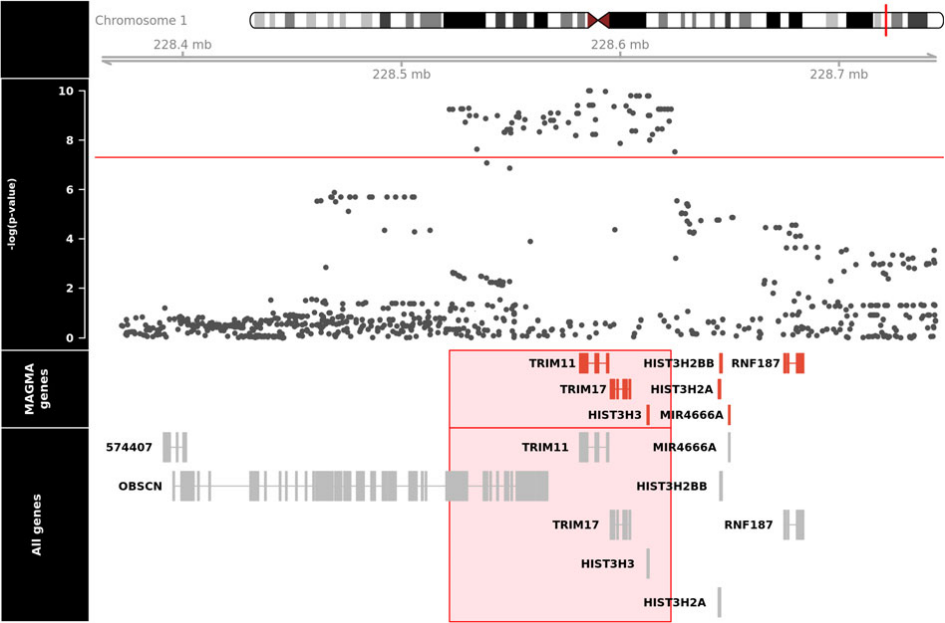
MAGMA analyses revealing significance at *TRIM11, TRIM17*, and *HIST3H3* genes. Gene boundaries are based on GRCh37/hg19. Red lines indicate the threshold for genome‐wide significance (*p* < 2.74 × 10^−6^). [Color figure can be viewed at http://wileyonlinelibrary.com]

MAGMA analyses (Fig 3) revealed that 4 genes passed genome‐wide significance in analyses run with and without 35kb upstream and 10kb downstream of each gene (*TRIM11, p* = 5.64 × 10^−9^; *TRIM17, p* = 8.99 × 10^−9^; *HIST3H3, p* = 1.29 × 10^−8^; *LOC101927401, p* = 5.72 × 10^−8^). *LOC101927401* appeared only in NCBI annotation and was absent in the queried gene expression databases; thus, it was excluded in downstream analyses.

**Figure 4:**
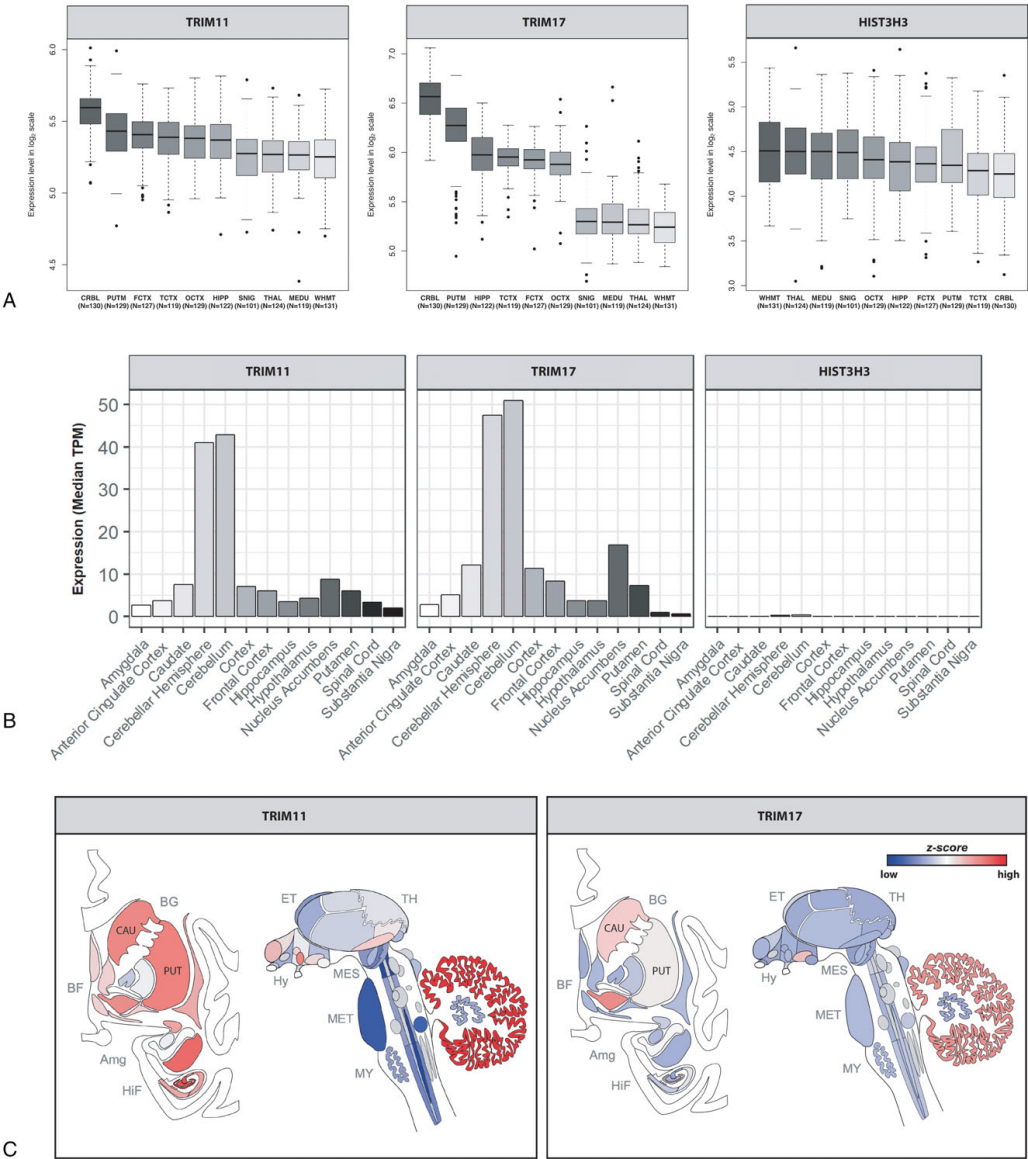
*TRIM11*, *TRIM17*, and *HIST3H3* brain expression in 3 databases. (A) BRAINEAC database. CRBL = cerebellum; FCTX = frontal cortex; HIPP = hippocampus; MEDU = medulla; OCTX = occipital cortex; PUTM = putamen; SNIG = substantia nigra; TCTX = temporal cortex; THAL = thalamus; WHMT = white matter. (B) GTEx database. (C) Allen Atlas database (Caucasian subjects). Amg = amygdala; BF = basal forebrain; BG = basal ganglia; CAU = caudate; ET = epithalamus; HiF = hippocampal formation; Hy = hypothalamus; MES = mesencephalon; MET = metencephalon; MY = myelencephalon; PUT = putamen; TH = thalamus; TPM = Transcripts Per Kilobase Million. Image credit: Allen Institute.

We then used human brain gene expression and coexpression data from the BRAINEAC, GTEx, and Allen Atlas databases to assess the expression profiles of genes identified in our MAGMA analysis: *TRIM11, TRIM17*, and *HIST3H3*. All 3 datasets revealed high levels of *TRIM11* and *TRIM17* expression in the brain, particularly cerebellum and putamen, whereas HIST3H3 expression appeared to be at the lower limit of detection in human brain (Fig 4).

We explored the cellular specificity of *TRIM11*, *TRIM17*, and *HIST3H3* expression in human brain using data provided by the Brain RNA‐Seq database. This demonstrated higher neuronal expression (n = 1) of *TRIM11* (0.38FPKM) compared to both *TRIM17* (0.12FPKM) and *HIST3H3* (0.10FPKM), which was expressed at the lower limit of detection. In comparison to its neuronal expression, *TRIM11* expression in glial cell types was lower (mature astrocytes, 0.14 ± 0.02FPKM, n = 12; microglia, 0.12 ± 0.02FPKM, n = 3; oligodendrocytes, 0.11 ± 0.01FPKM, n = 3). We also explored cell‐specific expression of the *TRIM11* and *TRIM17* mouse orthologues across the brain using single cell RNA‐Seq data (http://dropviz.org/) generated from mouse brain tissue. These data suggested that expression of *TRIM11* was highest in the spiny projection neurons (SPNs) of the striatum, with high expression in SPNs of both the “direct” and “indirect” pathways. In contrast, *TRIM17* expression was generally lower, with the highest expression detected in the neurons of the substantia nigra.

Our colocalization analysis did not reveal any significant associations between our GWAS signals and eQTL data in the BRAINEAC and GTEx databases in all brain regions. However, of note, expression analyses using GTEx data revealed that several SNPs from the chromosome 1q42.13 locus reaching genome‐wide significance in our GWAS were significant eQTLs for *TRIM11* and *TRIM17* in skin and thyroid tissues when analyzed individually.

### *Discussion*

To our knowledge, this is the first GWAS of clinical phenotype in PSP. We show that variation at the chromosome 1q42.13 locus determines clinical phenotype in PSP with a very strong effect size (odds ratio = 5.5). The validity of our GWAS results is increased by similarly sized association signals and minor allele frequencies being observed in 2 independent cohorts, with a genome‐wide significant association achieved when the 2 cohorts were combined. Furthermore, when considering the subset of MDS criteria–phenotyped cases from the original PSP case–control GWAS, RS and non‐RS group MAFs of directly genotyped SNPs in high LD with our lead SNP are supportive of our findings. We are also reassured by our PD genotyping data, which revealed that our GWAS signal would have been attenuated if PD cases had been inadvertently included in our clinically diagnosed non‐RS group. However, this is unlikely, as we only included PSP‐P and PAGF cases that fulfilled probable PSP MDS criteria for these phenotypes. We suspect that none of our significant SNPs reached genome‐wide significance in the original PSP case–control GWAS because pathologically diagnosed PSP cases would have contained a mixture of RS and non‐RS cases. When considering our data, we can infer that the combined MAFs of RS and non‐RS cases would have resulted in overall PSP group MAFs that were similar to those of healthy controls. The validity of our NeuroChip genotyping and imputation were confirmed by the additional genotyping we carried out to span the chromosome 1q42.13 locus. The validity of our independent cohorts is suggested by the following: (1) in both cohorts, a majority of cases with an initial non‐RS phenotype had a final clinical diagnosis of RS, as previously shown by other groups^28^; (2) our cohorts had similar MAFs for risk variants identified in the PSP case–control GWAS^15^; and (3) there was 100% concordance between clinical and pathological diagnoses in our clinical cohort for the subset of patients that had undergone postmortem examination.

MAGMA analysis confirmed signals in *TRIM11, TRIM17*, and *HIST3H3* genes. There was evidence for differential regional brain expression of *TRIM11* and *TRIM17*. Both human and mouse RNA‐Seq data revealed high levels of neuronal *TRIM11* expression. In addition, it is likely that our GWAS was significantly underpowered to detect signals in our eQTL colocalization analyses.

It is important to note that SNPs within this genomic locus are in high LD, as evidenced by the spread of genome‐wide significant SNPs identified in our GWAS (see Fig 2). Therefore, it is challenging to know which gene is driving our association signal. However, the localization of the lead SNP in our dataset and the gene expression profiles described above suggest that *TRIM11* is the most likely candidate gene at the chromosome 1q42.13 locus. The eQTL profile of our significant SNPs in GTEx, when analyzed individually, was particularly interesting. The strong association between several SNPs in high LD with rs564309 and decreasing *TRIM11* and *TRIM17* expression in nonbrain tissues highlight the concept of tissue/region/cell‐specific expression of transcripts potentially being determined by disease state and at specific time points in development or ageing.^29^ However, our data do not exclude potentially important functional roles for the other transcripts within this locus.

The major limitation of our study is that our cohort size was relatively small compared to case–control GWAS in PSP^15^ and other neurodegenerative diseases.^30^, ^31^ Further replication of our findings in larger cohorts is desirable, including other non‐RS phenotypes such as PSP‐F, to confirm the role of *TRIM11* and identify genetic determinants of clinical phenotype in PSP at other loci. Furthermore, our lead SNP is an intronic variant that does not pass the false discovery rate threshold for being a brain eQTL at the genes in our locus of interest, and the only coding variants that it is in LD with are from a gene (*OBSCN*) that is unlikely to be of biological relevance to PSP pathology. This is a common dilemma, as a majority of risk variants identified in GWASs over the past 2 decades are not associated with coding changes in expressed proteins.^32^ Furthermore, disease‐associated intronic SNPs can regulate the expression of more distant genes. When referring to BRAINEAC and GTEx, rs564309 was found not to be a brain eQTL at distant genes outside of our region of interest. Functional impacts of intronic variants may arise in modes other than gene expression, including via splicing and methylation patterns of targeted transcripts and proteins. It remains a challenge to understand the functional consequences of noncoding genetic variation linked to phenotype, and so functional studies are vital. However, gene expression studies in postmortem disease tissue can be challenging to interpret because of the confounding effects of changes on cell populations.^33^

TRIM proteins are biologically plausible candidates as determinants of clinical phenotype in PSP and promising targets for follow‐up functional studies. The TRIM family of proteins, most of which have E3 ubiquitin ligase activities, have various functions in cellular processes including intracellular signaling, development, apoptosis, protein quality control, autophagy, and carcinogenesis.^34^ A recent study has shown that TRIM11 has a critical role in the clearance of misfolded proteins via the ubiquitin proteasome system (UPS), in this case pathogenic fragments of both ataxin‐1 (Atxn1 82q) and huntingtin protein (Httex1p 97QP).^35^ Other groups have shown that lysine residues of tau are targets for polyubiquitination, which induces proteolytic degradation of tau via the UPS.^36^ Furthermore, tau accumulation has been associated with decreased proteasome activity in mouse tauopathy models, suggesting a feedback loop between impaired protein degradation, aggravated by a protein aggregate–based impairment of proteostasis.^37^ These findings coincide with previous studies that have identified the UPS as a potential drug target in the treatment of neurodegenerative conditions.^38^, ^39^ Overexpression of TRIM17, partly controlled by glycogen synthase kinase 3 pathways, has been shown to initiate neuronal apoptosis in cell models.^40^ This was later shown to be mediated by increased degradation of the antiapoptotic protein, myeloid cell leukaemia 1 (Mcl‐1), via the UPS.^41^

Based on our data, we hypothesize that common variation at the chromosome 1q42.13 locus modifies the function of TRIM11 to varying degrees in specific brain regions. In the more slowly progressing non‐RS syndromes, an increase in TRIM11 function may lead to increased degradation of toxic tau species via the UPS, therefore protecting against tau pathology. Conversely, a decrease in protein function in the brainstem is more likely to promote rapid accumulation of tau aggregates, manifesting as the malignant RS phenotype of PSP.

In summary, the results of this study suggest that common variation at the *TRIM11* locus may be a genetic modifier of clinical phenotype in PSP. Our findings add further evidence for the UPS playing a key role in tau pathology and therefore representing a potential target for disease‐modifying therapies. Further GWASs with larger cohorts to confirm our findings and identify other genetic signals, screening of whole genome/exome sequencing data for rare variants in *TRIM11*, and follow‐up functional studies at this locus are priorities.

## Author Contributions

Study concept and design: E.J. and H.R.M.; data acquisition and analysis: E.J., J.W., M.M.X.T., M.S., A.P., R.F., K.Y.M., D.Z., R.H.R., R.d.S., M.‐J.G., G.R., U.M., S.A.‐S., S.M.G., T.R., and J.L.H.; drafting and critical analysis of manuscript: E.J., A.J.L., T.T.W., J.H., T.R., G.U.H., J.L.H., M.R., and H.R.M.

## Potential Conflicts of Interest

Nothing to report.
